# Remission of Childhood-onset Epilepsy

**DOI:** 10.15844/pedneurbriefs-29-1-3

**Published:** 2015-01

**Authors:** Jena M. Krueger, Douglas R. Nordli

**Affiliations:** 1 Division of Neurology, Ann & Robert H. Lurie Children's Hospital of Chicago, Chicago, IL; 2 Departments of Pediatrics and Neurology, Northwestern University Feinberg School of Medicine, Chicago, IL

**Keywords:** Epilepsy, Epidemiology, Prognosis

## Abstract

Investigators from the Ann and Robert H. Lurie Children's Hospital of Chicago, Chicago IL and the Yale School of Medicine, New Haven, CT have conducted a prospective cohort study of 613 children with newly diagnosed epilepsy to evaluate the occurrence of complete remission and predictive factors. Of the 613 patients recruited, 516 were followed for greater than 10 years and of those, 328 (63.5%) attained complete remission.

Investigators from the Ann and Robert H. Lurie Children's Hospital of Chicago, Chicago IL and the Yale School of Medicine, New Haven, CT have conducted a prospective cohort study of 613 children with newly diagnosed epilepsy to evaluate the occurrence of complete remission and predictive factors. Of the 613 patients recruited, 516 were followed for greater than 10 years and of those, 328 (63.5%) attained complete remission. Of the patients that achieved complete remission, 23 (7%) experienced a relapse while 311 patients remained in complete remission at the end of follow up. Relapse was more likely to occur within the first five years. There were no relapses reported after 10 years of complete remission. Multivariable analysis indicated focal self-limited epilepsy, uncharacterized epilepsy and uncomplicated epilepsy patients were more likely to achieve complete remission. Patient's with an age of onset equal to or greater than 10 years or patient's with early school or developmental problems were less likely to achieve complete remission. Remission achieved by 2 years after onset was associated with a higher probability of complete remission, while late relapse or pharmacoresistance decreased the chances of complete remission. When patients were divided into uncomplicated (normal neurological and imaging exams, absence of clear intellectual disability and no identified insult or condition to which epilepsy could be ascribed to) and complicated (one or more of the previously listed factors) categories, older age of onset remained a risk factor to decrease the chance of complete remission in both groups. In addition, in uncomplicated patients, early school problems or developmental delays and a family history of epilepsy were found to decrease the chance of achieving complete remission. In the complicated group, imaging abnormalities and intellectual disabilities were found to decrease the chance of complete remission. [1]

COMMENTARY. The International League Against Epilepsy has delineated epilepsy as “resolved” after seizure freedom for 10 years, with at least 5 years off medication in an official report in January 2014 [2]. Multiple studies, including the Mayo Clinic Record Linkage Study, the British Tonbridge Study and the National General Practice Study of Epilepsy have noted that over half of children will reach complete remission and fall in the category of “resolved” epilepsy [3]. The Tonbridge study reports up to 80% of patients have resolved epilepsy by 20 years, although this sample is derived from general practitioners and may be biased toward a less complicated population when compared to other studies.

Despite positive data regarding the resolution of epilepsy, a review of Dutch, Japanese, and Canadian studies by Camfield and Camfield, in Halifax, Nova Scotia, illustrates that seizure resolution is not necessarily equivalent to a cure, and those in remission may have poor lifestyle outcomes [4]. Even patients with recorded normal intelligence have lower markers of social outcome (including failure to complete high school, pregnancy outside of a stable relationship, never in a romantic relationship >3 months, depression or another psychiatric diagnosis, unemployment, living alone and poverty).

Further research will be needed in the future to determine if early epilepsy factors can be modified to improve long term outcomes, including seizure resolution and lifestyle outcomes.
